# Editorial: Bio-inspired Audio Processing, Models and Systems

**DOI:** 10.3389/fnins.2019.00978

**Published:** 2019-09-13

**Authors:** Shih-Chii Liu, John G. Harris, Mounya Elhilali, Malcolm Slaney

**Affiliations:** ^1^Institute of Neuroinformatics, University of Zurich and ETH Zurich, Zurich, Switzerland; ^2^Department of Electrical & Computer Engineering, University of Florida, Gainesville, FL, United States; ^3^Department of Electrical and Computer Engineering, Whiting School of Engineering, Johns Hopkins University, Baltimore, MD, United States; ^4^Google, Mountain View, CA, United States

**Keywords:** neuromorphic audio sensors, spatial hearing, auditory perception, inter-aural time delay, spike-based computation, spatial-temporal response fields, attention decoding, saliency

## Introduction

Bio-inspired systems look at biology to inspire engineering solutions that help explain, emulate and complement the intricate processes that take place in a biological system. As such, they operate at the intersection of biology and engineering and leverage advantages from both disciplines. When applied to brain sciences, bio-inspired systems often use non-conventional approaches to solve complex sensory and cognitive tasks.

Recent developments in sensor design, algorithmic configurations, and network-level processing show the promise and efficacy of brain-like systems in solving complex tasks. While vision systems are widely explored in neuromorphic engineering design, audio systems offer unique challenges. These include careful handling of the time and space dimensions, issues related to temporal sampling and signal representation in both time and frequency, leveraging the redundancy in audio signals for complex detection and recognition tasks, as well as robust processing against noise and other interferers and maskers.

Our auditory systems have evolved highly efficient solutions to audio scene analysis, spatial understanding, and sound recognition. We wish to better understand the biological solutions that allow the brain to process sounds in unknown and highly distorted conditions; in order to help advance state-of-art audio systems that often operate well under well-controlled environments but fail to generalize, adapt and efficiently process unknown conditions. Furthermore, we want to apply engineering methods to better understand biological processes, using non-invasive methods. By leveraging both our knowledge of the biology in building better systems, as well as new technological advantages to unravel secrets of the brain, we hope to enrich the conversation across both disciplines in order to advance our understanding of the brain function and help improve technologies that impact our lives in a wide range of domains.

## Overview

This special topic issue describes the latest advances in research on sensors, models, networks, and hardware for audio processing, hearing systems, and speech technologies. Broadly speaking, the papers in this special issue fall into four broad classes:

1. Bio-inspired implementations
2. Models based on spikes
3. Sound recognition
4. Attention decoding.

Bio-inspired systems often start with hardware designed to mimic and/or capitalize on the advantages of biological systems. With regards to processing acoustic cues, a paper by Xu et al. describes a digital hardware FPGA implementation of a well-known CAR-FAC cochlear model that mimics the auditory physiology seen in the biological cochlea. Similarly, our auditory system is exquisitely sensitive to the differences in signals received between the two ears. The paper by Isbell and Horiuchi explores how the auditory system might change the timing of pulses in an echo-location system. Finally a paper by Encke and Hemmert introduces a spiking neuron model based on recent physiological findings in mammals for the detection of interaural time differences for sound localization.

The most obvious difference between conventional solutions to auditory processing and biological systems is the way that our biology depends on discrete spikes to represent the sensory signal. Toward this end, papers by Anumula et al. and Acharya et al. investigate different ways to represent the spiking information in ways amenable to conventional machine-learning methods. The paper by Wu et al. takes these approaches to feature discovery a step further by using a self-organizing network to design the best feature representation. Then, the paper by Li and Príncipe looks at ways to extend the temporal information using kernel methods that can choose the optimal representation.

An important task for the auditory system is to understand and identify the sounds around us. The paper by McWalter and Dau considers high-level features that combine information across time and frequency for synthesizing and perceiving auditory textures. A paper by Zuk et al. looks at how we perceive musical beats, comparing the information from bottom-up (sensory) processes vs. top-down (cognitive) expectations. Finally a paper by Huang et al. looks at ways to build models of what makes a sound salient in its environment.

To conclude this special issue, much effort recently has gone toward finding methods that allow us to monitor the attention of a user. In the visual world, the eyes provide an important clue, but no such obvious signal exists for the auditory world. The paper by Alickovic et al. summarizes several approaches based on regression and correlation analysis that allow us to match the audio signal and brain's response. Wong et al.'s paper adds further details on regularization methods for regression-based methods, which are needed to make the computations stable. To put it all together, a paper by Miran et al. builds an end-to-end solution that considers the statistics of the input signal and the output decision to build an optimal decoder of a user's attentional state.

We hope you find these 13 papers illuminating. They represent the state of the art in bio-inspired audio-processing models and systems.

## Author Contributions

This editorial was written and edited by MS, S-CL, ME, and JH.

### Conflict of Interest Statement

The authors declare that the research was conducted in the absence of any commercial or financial relationships that could be construed as a potential conflict of interest.

